# Six-Degree-of-Freedom Freehand 3D Ultrasound: A Low-Cost Computer Vision-Based Approach for Orthopedic Applications

**DOI:** 10.3390/diagnostics14141501

**Published:** 2024-07-12

**Authors:** Lorenzo De Sanctis, Arianna Carnevale, Carla Antonacci, Eliodoro Faiella, Emiliano Schena, Umile Giuseppe Longo

**Affiliations:** 1 Fondazione Policlinico Universitario Campus Bio-Medico, Via Álvaro del Portillo, 200, 00128 Rome, Italy; lorenzodesanctis1@gmail.com (L.D.S.); arianna.carnevale@policlinicocampus.it (A.C.); carla.antonacci@unicampus.it (C.A.); e.faiella@policlinicocampus.it (E.F.); e.schena@unicampus.it (E.S.); 2 Research Unit of Orthopaedic and Trauma Surgery, Department of Medicine and Surgery, Università Campus Bio-Medico di Roma, Via Álvaro del Portillo, 21, 00128 Rome, Italy; 3 Laboratory of Measurement and Biomedical Instrumentation, Department of Engineering, Università Campus Bio-Medico di Roma, Via Álvaro del Portillo, 21, 00128 Rome, Italy

**Keywords:** freehand 3D ultrasound, musculoskeletal ultrasound, probe tracking, ArUco markers, orthopedics

## Abstract

In orthopedics, X-rays and computed tomography (CT) scans play pivotal roles in diagnosing and treating bone pathologies. Machine bulkiness and the emission of ionizing radiation remain the main problems associated with these techniques. The accessibility and low risks related to ultrasound handling make it a popular 2D imaging method. Indeed, 3D ultrasound assembles 2D slices into a 3D volume. This study aimed to implement a probe-tracking method for 6 DoF 3D ultrasound. The proposed method involves a dodecahedron with ArUco markers attached, enabling computer vision tracking of the ultrasound probe’s position and orientation. The algorithm focuses on the data acquisition phase but covers the basic reconstruction required for data generation and analysis. In the best case, the analysis revealed an average error norm of 2.858 mm with a standard deviation norm of 5.534 mm compared to an infrared optical tracking system used as a reference. This study demonstrates the feasibility of performing volumetric imaging without ionizing radiation or bulky systems. This marker-based approach shows promise for enhancing orthopedic imaging, providing a more accessible imaging modality for helping clinicians to diagnose pathologies regarding complex joints, such as the shoulder, replacing standard infrared tracking systems known to suffer from marker occlusion problems.

## 1. Introduction

Musculoskeletal disorders are considered to be the leading worldwide cause of pain and physical impairment [1,2]. Among them, shoulder diseases (SDs) play a significant role, and patients who suffer from SDs may experience several consequences, such as reduced range of motion, worse quality of life, and severe restrictions in performing Activities of Daily Living [3,4,5]. The early diagnosis and treatment of musculoskeletal pathologies like osteoarthritis are essential to prevent additional joint damage and improve patients’ quality of life [3,4,5].

Medical imaging plays a crucial role in the diagnosis and treatment of musculoskeletal diseases [6]. The primary imaging techniques used for pathology diagnosis are X-rays, computed tomography (CT), magnetic resonance imaging (MRI), and ultrasound (US) imaging [7]. In X-ray and CT scans, the examination involves the patient being exposed to ionizing radiation, with a minimum of 0.02 mSv for a chest X-ray and 8.00 mSv for the corresponding CT scan [8]. The use of ionizing radiation requires the application of specific protocols and the execution of exams in shielded rooms to avoid exposing personnel, patients, and visitors to unnecessary doses [9]. MRI, even if it does not involve the emission of ionizing radiation, requires bulky instruments in dedicated spaces. Not all patients can undergo exams involving the intense magnetic fields that this technique uses [10]. US uses mechanical (sound) waves and their interaction with the tissues to produce an image [11]. This imaging system does not require the emission of ionizing radiation; it is considered a relatively safe technique in most situations, and there are commercially available devices that can be transported easily [12]. Standard B-mode US can output 2D cross-sections of the anatomical region scanned [13].

Freehand 3D US is an imaging technique that consists of manually moving a conventional 2D US probe over the area of interest to acquire a series of 2D images, which are then reconstructed into a three-dimensional representation. This approach relies on tracking the position and orientation of the probe, often using a tracking device or external sensor, to accurately assemble 2D slices into a 3D volume [14].

The first US probe optical tracking attempts were detailed in the literature as early as 1990 [15]. Today, infrared (IR) stereophotogrammetric optical tracking systems vary based on the number of cameras and the application. For example, compact systems with only two cameras are suitable for use in the operating room, whereas entire laboratories are employed for kinematic gait analysis [16,17]. These systems recognize only the marker as a generic point, and six-degree-of-freedom (6 DoF) tracking is possible only with a cluster of at least three rigidly attached markers. Indeed, 6 DoF tracking of an object might require more than two cameras, as the object itself could obscure the marker’s view from the camera, a phenomenon known as self-occlusion.

Several methods for probe-tracking are reported in the literature [18]. In particular, freehand 3D ultrasound localization systems may include optical tracking systems, mechanical devices, electromagnetic sensors, and acoustic systems [19,20,21]. The mentioned systems have some limitations: optical systems are prone to occlusion, mechanical systems are often bulky and heavy, electromagnetic systems have low positional accuracy and cannot be applied to all patients, and acoustic systems have non-negligible latency [22]. In the literature, an attempt to validate freehand 3D ultrasound with a low-cost approach is reported, using a single printable fiducial marker to capture the US probe trajectory, albeit without solving the occlusion problem and limiting the freedom of scanning [23].

This study aimed to implement a probe-tracking method utilizing a standard RGB camera, an identification-tracking algorithm based on computer vision (CV), and binary ArUco fiducial markers (consisting of a black square with a white pattern made of paper) [24]. The idea was to create a cluster of ArUco markers glued onto a dodecahedron as support and use an algorithm to assess the positions and orientations of the US images in the camera frame, applying the same principle used in [25] to track a ball pen. Attaching this cluster to the US probe makes it possible to calculate a rigid body transformation to obtain a volume filled with the acquired US images. This multiple passive marker method could help physicians to perform and interpret US scans of curved surfaces such as human joints by solving the occlusion problem of traditional optical systems.

## 2. Materials and Methods

The proposed approach’s goal was to map the pixels of an US image into a volume. It was, thus, necessary to assign, to each pixel i, a set of three coordinates ($x_{i},\;y_{i},\;z_{i}$) to define its position ($u_{i}$) in space relative to the camera reference frame as follows:

$$u_{i}={\left(x_{i},\;y_{i},\;z_{i}\right)}^{⊤}$$

**Figure 1:**
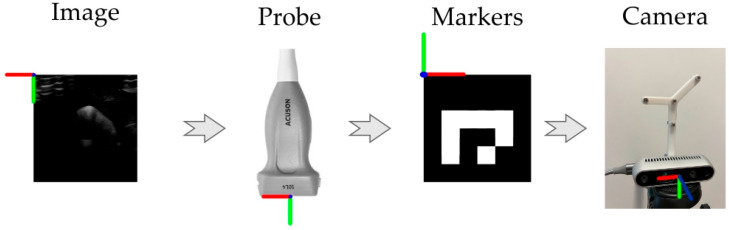
A representation of the frame transformations required to convert 2D image pixels in 3D camera coordinates. Reference axis colors: red (x-axis), green (y-axis), blue (z-axis).

Pixel coordinates were calculated through a concatenation of homogeneous transformation matrices. A rigid transformation in SE(3) is written in the form of a 4×4 matrix

$$T\in \;\mathrm{SE}(3):T=\left(\begin{array}{cc} R & t\\ 0 & 1 \end{array}\right)$$

where R is the rotation sub-matrix such that

$$R\in \left\{R\in M_{3,3}\left(R\right):\;R^{⊤}=R^{-1},\;det\left(R\right)=+1\right\}$$

and $t\in R^{3}$ is the translation vector [26,27]. To maintain dimensional correctness during multiplication, vectors u were expressed as points p=u1 so that $p^{\prime }=T$. Specifically, the US image pixels were transformed from the image reference frame to the probe virtual frame, from the probe frame to the marker cluster frame, and from the marker cluster frame to the camera frame, as shown in Figure 1.

**Figure 2:**
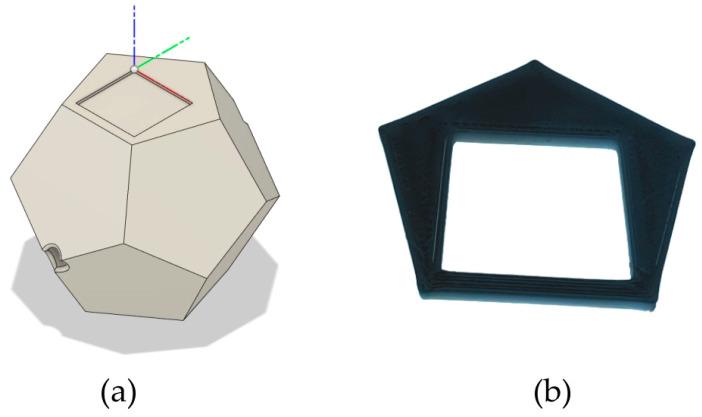

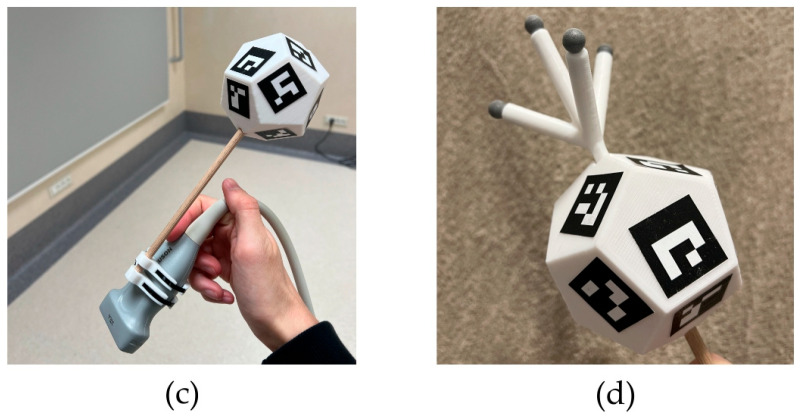
(**a**) Dodecahedron CAD design, (**b**) marker placement mask, (**c**) US probe fixture, and (**d**) removable IR cluster module.

For this study, a RealSense D435i (Intel Corporation Inc., Santa Clara, CA, USA) camera was used to obtain a video stream, but its depth capabilities have not been utilized in any way. The detection algorithm requires the marker to be attached to a planar surface. A regular dodecahedron was designed using a Computer-Aided Design (CAD) software (Fusion 360, version 2.0.18961, Autodesk Inc., San Francisco, CA, USA) and fabricated in white polylactic acid with the assistance of a 3D printer (Ender 3, Creality Co, Ltd., Shenzhen, China) (Figure 2a). A set of twelve 25 mm edge ArUco markers were printed on standard A4 paper using an office laser printer. Each of the twelve markers was cut out and glued using a dedicated mask (Figure 2b) onto one face of the dodecahedron with its side parallel to the dodecahedron edge at a 2.5 mm distance from it. A universal fixture was fabricated to attach the dodecahedron posteriorly to the US probe body (Figure 2c). At the meeting point between three edges, posteriorly, a cluster of IR markers was press fitted for tracking the probe with the reference technique (Figure 2d). To record the position of the IR markers, a set of 10 cameras was used (Qualisys, AB, Göteborg, Sweden).

**Figure 3:**
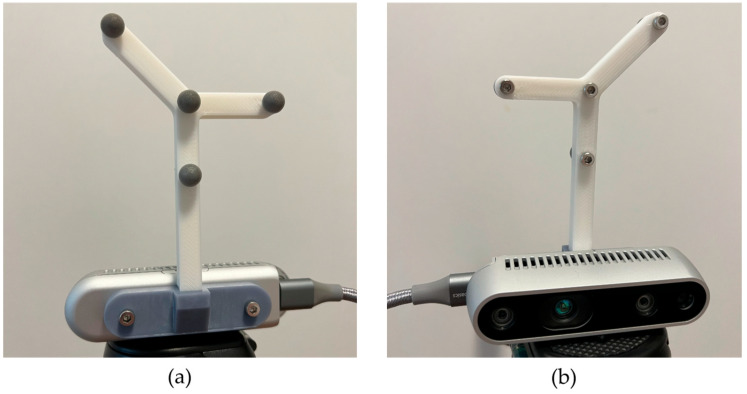
Camera IR marker cluster: (**a**) rear view and (**b**) front view.

Another IR marker cluster was placed on the camera using a dedicated fixture to calculate the homogeneous transformation between the motion capture system frame and the camera frame (Figure 3). The marker positions relative to the camera frame were extracted from the CAD software of the fixture and the technical documentation of the camera.

**Figure 4:**
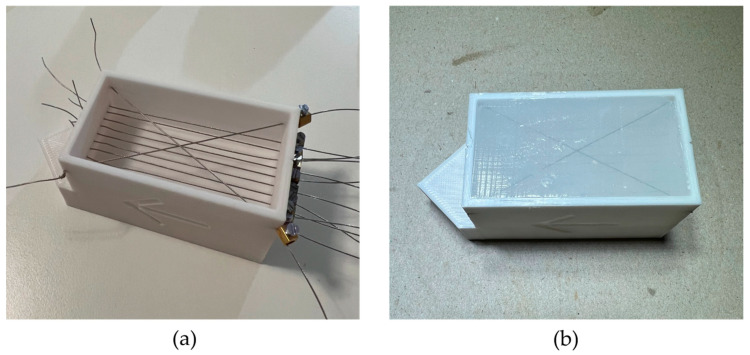
Calibrator phantom: (**a**) crossed-wire setup on multiple levels (**b**) phantom after silicone gel complete cross-linking.

To determine the required homogeneous transformation between the dodecahedron and the US probe and between the probe and the US video frame (i.e., probe calibration), a calibrator phantom was necessary [28]. Into a specifically designed container, two steel wires (Ø 0.5 mm) were crossed at the first level at a depth of 8 mm from the surface (proximal layer in Figure 4a). Below this level, at a depth of 18 mm, a second one consisted of ten parallel wires (Ø 0.5 mm) spaced 4 mm apart (distal layer in Figure 4a). Two tests were conducted to choose the following filling material (i.e., substrate): acetic silicone, shown in Figure 4b, and a 6.6% *w*/*w* agarose gel, as proposed by [29], mixed with benzalkonium chloride [30]. A mask that constrains the probe used to move perpendicular to the parallel wires was 3D printed and press-fitted onto the phantom.

The previously described calibrator was connected to a modular system consisting of two additional ArUco markers and an IR marker cluster. A linear transducer US probe (Acuson 10L4, Siemens Healthineers AG, Erlangen, Germany) was used.

**Figure 5:**
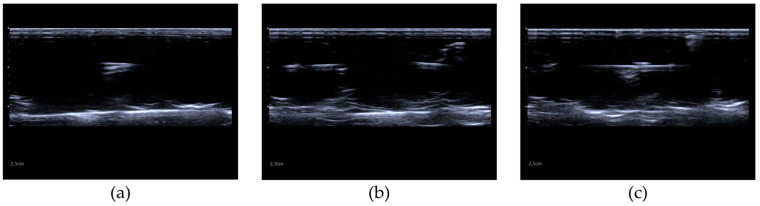

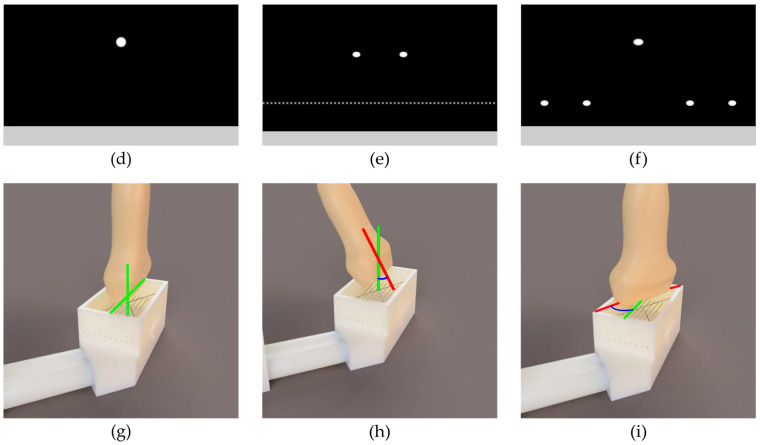
Probe calibration: (**a**) optimal case, (**d**) illustration, and (**g**) render; (**b**) pitch error, (**e**) illustration, and (**h**) render; and (**c**) roll error, (**f**) illustration, and (**i**) render. In the second row, slice thickness is assumed to be nearly zero to simplify the concept. In the third row, the angle in blue represents the rotation for which the above error is obtained.

Calibration was initiated when the operator, seeing only a single point (formed by the intersection of the two steel wires on the first layer) and the bottom of the container on the monitor, captured a snapshot of the tracking system marker trajectories and the US video stream. If multiple points or lines were present in the acquired US image, as shown in Figure 5, it would not be possible to reconstruct the calibration matrix because the calibrator landmarks (e.g., the intersection of the two wires on the first layer) would be lost. The calibration matrix was calculated as follows: first, the transformation from the dodecahedron frame to the centre of the two wires was found with the appropriate rotation, given by the formula

$$T_{Cam2Dod}^{-1}T_{Cam2Cal}=T_{Probe}$$

Knowing that

$$T_{Cam2Dod}T_{Probe}T_{Cam2Cal}^{-1}=I_{4}$$

and then the pixel size was determined; subsequently, the transformation between the origin of the US frame and the origin of the calibrator was found, characterized by a simple two-dimensional translation.

**Figure 6:**
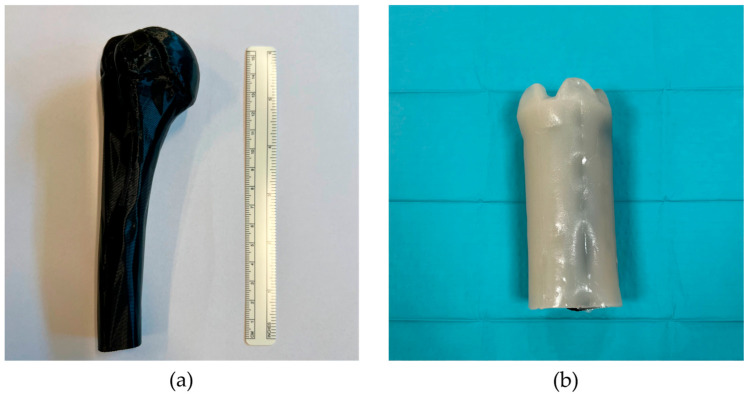
Phantom: (**a**) a 3D printed humerus model and (**b**) final agarose gel phantom.

To simulate a real US scan feeling, a phantom representing the humeral portion of the upper limb was made. The 3D model, segmented from an anonymized CT scan, is shown in Figure 6a. The bone model, made of PLA, was immersed in a 3% *w*/*v* agarose–water gel composed following the agar phantom operating standard procedure to mimic the soft tissue properties (Figure 6b) [31,32,33].

To capture and process data, a computer with a 6-core, 12-thread CPU (Ryzen 5 5600X, Advanced Micro Devices, Inc., Santa Clara, CA, USA), 16 GB of memory (Corsair Gaming, Inc., Fremont, CA, USA), and a 1TB NVMe solid-state storage was used. Factory camera calibration parameters were used to compute all data. After orienting the camera towards the scanning site and setting the acquisition parameters (capture duration, US machine’s region of interest, and starting delay), the software synchronized using Precision Time Protocol (PTPv2), the motion capture-dedicated workstation’s clock with the acquisition computer one, and started the IR recording [34,35]. The software then populated two arrays, respectively, with RGB frames from the camera, and their arrival timestamps until the capture interval condition was satisfied. Given the positions of each uniquely identified marker corner in the local frame (extracted from the CAD software) and having the coordinates of at least 4 points in the camera view, the dodecahedron pose was calculated by solving the Perspective-n-Point problem [36]. In this study, two tests were conducted: The first attempt consisted of computing the pose of the dodecahedron $T_{Cam2Dod}^{RGB}$ and concatenating it with the probe matrix $T_{Probe}$ obtained from the calibration phase (i.e., concatenation method), and the second one consisted of transforming all the corner coordinates in the probe frame and then directly computing the probe pose $T_{Cam2Probe}^{RGB}$ (i.e., direct PnP method). To accomplish those tasks, an initial solution was obtained via the method proposed in [37] using the RANdom Sample And Consensus (RANSAC) algorithm [38]. The set of corner vectors was augmented with additional ones generated by averaging the identified pairs of points. Assuming k as the number of detected markers, the additional points were calculated as follows:

$$u_{avg}=\frac{u_{i}+u_{j}}{2},\;\;\forall i,\;j=1,\;\ldots ,\;k\;\;|\;i\neq j$$

The inlier input points selected by the RANSAC algorithm were used to refine the initial pose using the Levenberg–Marquardt optimization scheme [39,40]. Additionally, the individual components of the translation vector t of each transformation matrix were filtered through the Savitzky–Golay method [41]. For each set of coordinates obtained from the IR system’s software (Qualisys Track Manager, version 2024.1, Qualisys AB, Göteborg, Sweden), knowing the respective coordinates in the dodecahedron frame, the optimal transformation matrix between the motion capture system frame and the dodecahedron frame was calculated using the Kabsch–Umeyama algorithm [42,43]. The motion capture *World to Dodecahedron* transformation was

$$T_{World2Dod}=T_{World2Cam}T_{Cam2Dod}^{IR}$$

where $T_{World2Cam}$ is the same among all frames given the static pose of the camera, while $T_{Cam2Dod}^{IR}$ varies.

Since the video sampling frequency of the RGB camera (60 Hz) was lower than the IR system’s one (200 Hz), data resampling (at 60 Hz) was needed: for the rotation sub-matrix, converted temporarily into quaternion form, the Spherical Linear intERPolation (SLERP) algorithm was used [44], while for translation vectors, linear interpolation was performed on each of the components. US reconstruction was beyond the scope of this article; thus, a set of 4 pixels representing the corners of a generic US image was considered. Assuming that the probe’s reference frame was coincident with the transducer geometric centre orthogonal projection on the probe surface, with the y-axis pointing in the direction of the US beam and the x-axis parallel with the transducer array direction, while also considering the Field of View (FOV) of the US probe to be 38 mm and the general useful depth d to be 30 mm, the corners of the image had the following coordinates:

$$\begin{array}{c} p_{c1}={\left(\frac{FOV}{2},\;0,\;0,\;1\right)}^{⊤},\;\;p_{c2}={\left(\frac{FOV}{2},\;d,\;0,\;1\right)}^{⊤}\\ p_{c3}={\left(-\frac{FOV}{2},\;d,\;0,\;1\right)}^{⊤},\;\;p_{c4}={\left(-\frac{FOV}{2},\;0,\;0,\;1\right)}^{⊤} \end{array}$$

For each acquired pose, the corners absolute coordinates were calculated using the reference matrix

$$p_{ref}=T_{Cam2Dod}^{IR}T_{Probe}p,\;\;\;\;p\in \left\{p_{c1},\;p_{c2},p_{c3},p_{c4}\right\}$$

using the direct PnP method matrix

$$p_{direct}=T_{Cam2Probe}^{RGB}p,\;\;\;\;p\in \left\{p_{c1},\;p_{c2},p_{c3},p_{c4}\right\}$$

and using the dodecahedron origin matrix

$$p_{concat}=T_{Cam2Dod}^{RGB}T_{Probe}p,\;\;\;\;p\in \left\{p_{c1},\;p_{c2},p_{c3},p_{c4}\right\}$$

To assess the accuracy and precision of the proposed system compared to the reference, the mean and standard deviation (STD) of the error were considered. Let N be the total number of corners analyzed (equivalent of the distributed pixels in the volume)

$$\varepsilon_{direct}=p_{direct}-p_{ref}$$

be the error of the direct PnP method and

$$\varepsilon_{concat}=p_{concat}-p_{ref}$$

be the error of the concatenation method, with $\bar{\varepsilon }$ as their relative mean and σ as their relative standard deviation. Thus, the following were considered:

$$\bar{\varepsilon }=\frac{1}{N}\sum \varepsilon ,\;\;\;\;\sigma =\sqrt{\frac{1}{N}\sum {\left(\varepsilon -\bar{\varepsilon }\right)}^{2}}$$

A total of four consecutive tests were conducted to evaluate the accuracy and precision of the proposed system. The experiments were performed under consistent conditions throughout the tests: the dodecahedron was not repositioned on the US probe, and one probe calibration sequence was completed before conducting the tests. The same operator performed all experiments on the same phantom. The IR system was calibrated following the standard procedure. The scan on the model was performed longitudinally, crossing over the same spot twice.

## 3. Results

Numerical results of the four experiments are presented in Table 1. For easier data interpretation, the Euclidean norm of the mean error and the STD were also reported.

**Figure 7:**
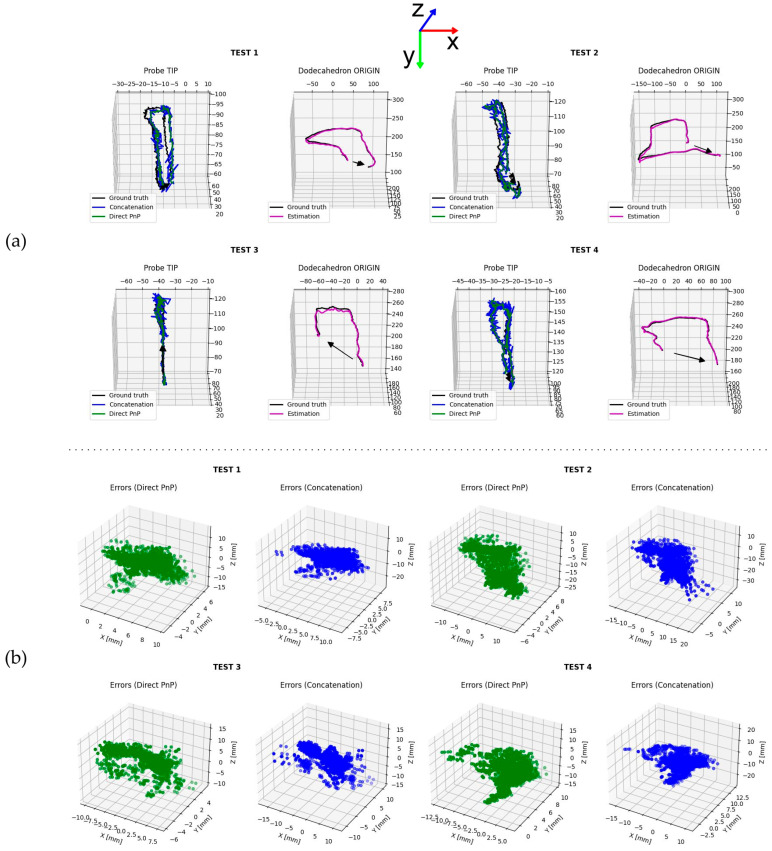
Plots: (**a**) probe tip and dodecahedron origin trajectories during all acquisitions and (**b**) probe tip trajectory error scatter plots. Axes are not equally scaled for visualization purposes.

Test 3 yielded the lowest STD norm of 5.534 mm, with a mean error norm of 2.858 mm, using the direct method. The concatenation method, applied to the same data, produced an STD norm of 6.290 mm, with a mean error norm of 2.777 mm. Dodecahedron origin and probe trajectories were plotted and are shown in Figure 7a, whereas comparisons between direct and concatenation methods are presented in Figure 7b using the scatter plots of the errors.

## 4. Discussion

Starting from a video camera and attaching ArUco markers to a dodecahedron, fixed to an US probe, it was possible to reconstruct the trajectory of the instrument using a low-cost system. The results were compared to an infrared optical tracking system considered to be the reference. The choice of using the RealSense D435i stereo camera was exclusively dictated by the possibility of having extensive technical documentation, a factory camera calibration, and mechanical drawings of the device. The regular dodecahedron, among all platonic solids, has a high number of faces, allowing the simultaneous detection of multiple ArUco markers, and it has a regular pentagon as its faces, permitting a maximum marker size equal to the edge length. Multiple-marker detection was needed to discern eventual pose ambiguities generated during the PnP problem resolution. The marker size choice was a compromise between visibility and the compactness of the dodecahedron: a larger marker makes detection simpler but also increases the dodecahedron’s dimensions, making it bulkier and uncomfortable to manipulate. Regarding the calibrator, the 4 mm spacing between the wires in the second layer is greater than the average slice thickness described for a linear probe but adequate to prevent artifacts caused by the beam shape [45,46]. The agarose substrate of the calibrator allows a sound velocity of 1571 ± 12 m/s compared to that of water, which is 1496 m/s at 25 °C [47]. The sound velocity in silicone corresponds to 1030 ± 60 m/s, which is significantly lower than that of water. This makes it not an ideal material for the described application [48]. The low deformation of both materials allowed us to maintain the probe at a constant distance of 8 mm from the first level and 18 mm from the second. The silicone phantom demonstrated much more solidity than the agarose one.

In this type of application, the main causes of artifacts are the distance between the camera sensor and the dodecahedron, camera calibration, and detection algorithm. The results in Figure 7a highlighted an inverse correlation between the z-axis distance of the markers from the camera sensor plane and the trajectory accuracy. Even with sub-pixel marker detection, the features are shrunk in the image as the object moves away from the sensor depending on the focal lengths of the camera, thus making the pose estimation less accurate. Furthermore, camera calibration, not mentioned in this work given the factory calibration used, is crucial; indeed, the lens manifests optical distortion affecting pixels that are further away from the optical centre of the camera. This phenomenon can be seen especially in Test 2 (Figure 7a), where the dodecahedron origin trajectory gradually shifts from the ground truth as the origin moves away from the origin in the −x direction. Fine-tuning the marker detection and pose refining algorithms can improve the accuracy and precision of the system. The parameters were adjusted empirically by running the elaboration script on Test 1 data until the optimal configuration was found (i.e., local minima). Considering the direct PnP and the matrix concatenation method, the two approaches are comparable, if the error mean norm is considered. The direct method features a lower error standard deviation norm because of the Savitzky–Golay translation vector filtering. US imaging provides only a limited view of the cortical bone. Therefore, a wide range of trabecular bone pathologies, especially osteoporosis, cannot be diagnosed through US [49]. It is necessary to specify that the US technician plays a crucial role in the scan execution: moving the probe slowly with a constant velocity and avoiding rapid changes in orientation drastically changes the reconstruction result [50]. In this work, the phantom was static in the camera frame, but in a real-world application, the patient could move, shifting pixel positions, thus making the scan uninterpretable. The static model used in the experiments did not fully simulate the dynamic changes in clinical environments. Future studies may take into account dynamic changes as these could affect image quality and the following interpretation. Robotic manipulators that have the potential to improve scan quality and repeatability by accommodating patients’ unique anatomical differences could replace the operator in the future [51]. Finally, it should be considered that the error measure defined to study the accuracy and precision of the proposed system is relative to the IR system taken as a reference. IR measurements also have inherent uncertainty, with respect to the actual position value, that could affect the obtained results. Compared with IR optical systems, although less accurate, the proposed system is not subject to self-occlusion since the ArUco marker is uniquely identified. Compared with electromagnetic and acoustic systems, the markers in the proposed system are passive and do not emit waves of any kind, and the latency problem is not present. Compared with mechanical systems, the proposed approach is lightweight since the dodecahedron fill percentage can be adjusted and does not require the use of bulky arms or mechanical components [22].

## 5. Conclusions

Both elaboration methods have demonstrated comparability, both among themselves and with respect to the reference, giving validity to the proposed capture system. Without the need to perform the dual recording (both with infrared and RGB system) carried out in this work for validation purposes, it could be possible to watch in real time the scanned region. Implementing deep learning algorithms, which have the potential to entirely replace tracking systems, including the one proposed in this study, could help to align captured US frames, thus reducing the error [52]. In the future, a refinement of the algorithm could further improve the accuracy and precision of the system, making it more reliable and useful. Given the results produced by the proposed approach, its effectiveness will be further investigated by involving real patients in a clinical study.

## Figures and Tables

**Table 1:** The numerical results of the performed recordings.

Method	Test #	$\bar{\varepsilon }$ [mm]			$\left\|\bar{\varepsilon }\right\|$ [mm]	σ [mm]			$\left\|\sigma \right\|$ [mm]
		*x*	*y*	*z*		*x*	*y*	*z*	
Origin ^1^	1	0.197	0.663	−0.045	0.693	2.655	2.686	3.312	5.024
	2	−0.135	1.734	−3.149	3.597	4.352	2.184	7.550	8.241
	3	−0.181	1.765	1.464	2.301	1.110	1.219	4.206	4.518
	4	−1.712	0.611	4.020	4.414	2.992	2.216	3.427	5.061
Direct	1	3.947	0.895	0.792	4.124	1.899	2.020	5.005	5.722
	2	0.928	0.599	−2.934	3.135	4.352	2.184	7.550	8.984
	3	−0.857	0.453	2.689	2.858	2.908	1.920	4.299	5.534
	4	0.675	3.860	0.373	3.937	2.206	1.773	5.817	6.469
Concatenation	1	3.893	0.928	0.629	4.051	2.578	2.478	5.965	6.954
	2	0.964	0.589	−2.797	3.017	4.860	2.678	7.643	9.445
	3	−0.846	0.505	2.596	2.777	3.539	2.496	4.561	6.290
	4	0.884	3.581	1.348	3.927	3.296	2.342	6.792	7.905

^1^ For the dodecahedron origin, data are obtained by multiplying by the null element in $SE\left(3\right)$, and it does not consider the rotational component of the last transformation matrix.

## Data Availability

Data sets generated during the current study are available from the corresponding author upon reasonable request.
